# Closing the implementation-evidence gap using data science: a transferable workflow applied to medical cost analysis

**DOI:** 10.3389/frhs.2026.1742807

**Published:** 2026-06-09

**Authors:** Fengyi Gao, Siew-Pang Chan

**Affiliations:** 1 Department of Medicine, Yong Loo Lin School of Medicine, National University of Singapore, Singapore, Singapore; 2 Institute of Geriatrics and Active Ageing, Tan Tock Seng Hospital, Singapore, Singapore

**Keywords:** behavioural medicine, CFIR, data science, medical costs, smart medicine

## Abstract

Rising healthcare expenditure is a global challenge, particularly in ageing populations with increasing chronic disease burden. While many studies identify cost determinants, few provide a reproducible analytic workflow that is transferable across health systems. This study presents a data science–based analytic pipeline to model medical expenditure, demonstrated using a large, publicly available health expenditure dataset. The pipeline incorporates feature engineering, statistical and machine learning models, model comparison, and interpretability analysis. The primary contribution is methodological, emphasising transparency, reproducibility, and potential transferability rather than context-specific conclusions. Although implementation science frameworks inform the interpretation, they are used only as conceptual lenses, and no implementation strategies are evaluated. The findings are therefore intended as forward-looking considerations, illustrating how data-driven approaches may inform future policy design, stakeholder engagement, and technology-enabled strategies for preventive care and chronic disease management, subject to local adaptation.

## 1 Introduction

This paper provides an empirical demonstration of a transferable data science workflow designed to support medical cost containment in Singapore, taking into account the nation's healthcare infrastructure and technological readiness. The United States Medical Expenditure Panel Survey (MEPS) is used for methodological illustration. A range of statistical techniques and machine learning methods are applied to demonstrate how structured data integration, feature engineering, predictive modelling, and cost decomposition can be systematically operationalised.

The objective is not to infer US-specific cost parameters for Singapore, but to showcase a scalable analytic pipeline that can be adapted to Singapore's administrative and claims-based datasets. By illustrating the workflow on a well-documented, high-quality panel dataset, this study clarifies the methodological architecture required to translate data into actionable cost-containment insights within a technologically advanced healthcare system.

Building on the empirical findings, this study highlights potential considerations relevant to Singapore's digital platforms for monitoring and accessing community health services. To situate these observations within an implementation perspective, the discussion draws on established frameworks as conceptual lenses only, without empirically operationalising them. These perspectives are intended to provide a structured basis for reflecting on future implementation and adoption considerations. In the Singapore context, they may offer insights relevant to ongoing national initiatives such as Healthier SG and the Healthy 365 app, particularly in relation to behavioural and system-level factors in digital health integration.

In addition, the study highlights considerations that may be relevant to family physicians when reflecting on the integration of behavioural medicine principles into community-based care. By conceptually linking predictive analytics with behavioural insights, the analysis identifies factors that may be associated with intervention uptake, medication adherence, and lifestyle modification. These observations are exploratory in nature and do not reflect implemented strategies; rather, they are intended to inform future research and consideration of technology-enabled approaches in smart medicine.

To support evidence-informed decision-making, this study brings together data science with insights from behavioural and implementation science as a conceptual perspective. The contribution is primarily methodological and is intended to inform future applications, policy considerations, and research directions rather than to evaluate implementation efforts. The findings highlight how such an approach may be relevant to improving healthcare delivery efficiency and informing long-term cost containment. In the Singapore context, the workflow may offer considerations aligned with ongoing shifts from reactive, treatment-focused care towards more proactive and preventive population health strategies, taking into account the country's digital infrastructure and technological readiness.

## 2 Background & conceptual framework

Medical inflation is a global phenomenon. Healthcare costs have remained persistently high in recent years as increasing demand for medical services places mounting pressure on health systems worldwide (1). Ageing populations further contribute to rising expenditure. According to the World Health Organization, the proportion of the global population aged 60 and above is projected to nearly double from 12% in 2015 to 22% by 2050 (1).

For Singapore, this represents a major public health and fiscal challenge. The core research question of this project is therefore: how can rising medical costs be contained effectively and efficiently within Singapore's existing healthcare system, infrastructure, and policy environment? Addressing this question requires a comprehensive examination of personal, institutional, and national determinants of healthcare expenditure in the context of Singapore's healthcare initiatives and socioeconomic development (2). A multi-faceted approach is required—one that integrates digital technology, infrastructural development, and cost-management strategies within Singapore's healthcare landscape (3).

Given the absence of a single publicly accessible dataset in Singapore that comprehensively captures healthcare utilisation, insurance coverage, and expenditure, this project employs data from the US MEPS as a methodological proxy. The objective is to identify, evaluate, and forecast key drivers of medical costs using advanced statistical and machine learning techniques. In addition, the project explores how the adoption of emerging technologies and behavioural medicine principles may contribute to sustainable cost management.

To reduce long-term medical expenditure, the study calls for strengthened public–private partnerships that prioritise prevention. Expanding telehealth services may reduce unnecessary hospital visits, while reinforcing primary care could prevent costly emergency treatments (4). Polyclinics and community health centres must be adequately resourced to alleviate pressure on tertiary hospitals. Greater hospital automation and AI-assisted diagnostics may reduce institutional overhead and improve efficiency, consistent with Singapore's Smart Nation agenda. Beyond mobile health services and AI-driven chatbots, broader public acceptance of advanced technologies is essential to deliver timely advice and personalised health coaching (5).

Singapore must confront the structural drivers of sustained medical inflation. However, comprehensive national-level data remain limited. This is partly due to strict personal data protection regulations under the Personal Data Protection Act (6), concerns about misinformation and public distrust, limited open-data culture, and a policy preference for controlled access to sensitive information (7). While such prudence safeguards privacy, it also constrains large-scale empirical research.

Accordingly, the MEPS is utilised as an illustrative dataset. Although not fully representative of Singapore's financing system, it enables the identification of important variables of total healthcare expenditure in an affluent society facing demographic ageing. The analysis applies a range of quantitative statistical and machine learning techniques, promoting the practice of the “3Cs” in Data Science—compare, contrast, and consolidate—while advancing the application of rigorous analytical tools in professional practice. Notably, several of the proposed data science techniques were not employed in the original MEPS analyses. The combination of data and methods may help generate actionable evidence to inform the future refinement and implementation of national and institutional cost-containment programmes.

From an implementation perspective, the discussion draws conceptually on the Consolidated Framework for Implementation Research (CFIR) as a structured lens for considering how the study's findings might inform future translation into practice. In this context, CFIR is recommended solely as an interpretive framework to guide reflection on potential implementation pathways, rather than as an empirically applied analytic framework within the present study. Note that CFIR highlights several domains relevant to future implementation, including intervention characteristics, the outer and inner settings, characteristics of individuals, and the implementation process. Within this perspective, the analytics-driven approach demonstrated in the present study illustrates the potential role of data-driven evidence in supporting cost-containment strategies. In the Singapore context, national priorities such as ageing populations and healthcare sustainability provide a relevant outer setting, while the country's technologically advanced healthcare system may facilitate digital integration. Considerations related to professional and patient engagement, as well as pilot testing and iterative refinement, may be important for future implementation.

Behavioural medicine complements this strategy. Lifestyle, psychological factors, and health behaviours significantly influence both individual and population health outcomes (8). Effective behavioural interventions hold strong potential for preventing disease, improving treatment adherence, and modifying risk trajectories (9). Therefore, scalable and sustainable interventions must be developed across diverse communities (10). Technology plays a central enabling role in this effort, and policymakers should prioritise the responsible adoption of advanced digital tools at the individual and system levels.

By integrating data science, behavioural medicine, and implementation science, this project seeks to advocate evidence-based interventions that enhance healthcare delivery and contribute to long-term cost containment. It offers a structured pathway for transitioning Singapore's healthcare system from reactive treatment towards proactive, preventive, and technology-enabled care.

## 3 Data & methods

Data from the MEPS were used for methodological illustration. MEPS is a nationally representative panel survey conducted in the United States since 1996 and administered by the Agency for Healthcare Research and Quality (AHRQ). The dataset is publicly accessible through the AHRQ website, and no additional ethical approval is required for secondary analysis of the de-identified data.

MEPS collects detailed information on households, individuals, employers, and healthcare utilisation, including visits to physicians, hospitals, pharmacies, and other medical providers. A complex, multi-stage probability sampling design is employed, with respondents followed over a two-and-a-half-year period across five interview rounds. This structure enables longitudinal tracking of healthcare utilisation, expenditure, and insurance coverage.

The sample comprised 3,064 individuals, of whom 2,955 were classified as healthcare users (i.e., individuals with positive medical expenditures). The target population primarily included US citizens aged 65 years and above covered by Medicare. The dataset includes variables on healthcare utilisation frequency, expenditure, sources of payment, and insurance status. Demographic and socioeconomic characteristics—such as age, gender, race, income, employment, education, and poverty status—are also available. Health conditions, access to care, satisfaction, and insurance coverage are comprehensively documented. Missing expenditure data are imputed using a weighted sequential hot-deck procedure to minimise bias. Expenditure categories include hospital care, office-based visits, dental services, prescription medications, vision aids, and medical supplies; over-the-counter medications and alternative therapies are excluded. The primary aim of this study is to demonstrate a transferable analytic workflow rather than to produce nationally representative population estimates. Accordingly, survey weights were not applied in the data analyses, as the focus was on quantitative modelling and methodological illustration.

MEPS consists of multiple components. The Household Component (HC) captures individual- and family-level data. The Insurance Component (IC) gathers information from employers regarding health insurance offerings. Healthcare utilisation measures include counts of medical visits, while cost variables encompass out-of-pocket spending, private insurance payments, Medicare, Medicaid, and other public sources. Insurance coverage categories include private, public, and uninsured status.

The MEPS complex survey design elements—survey weights, strata, and primary sampling units—were not incorporated into the analyses. All analyses were carried out with the unweighted dataset. This choice was made because the primary objective of the paper is methodological demonstration rather than population-level estimation. Specifically, we aim to present a transferable analytic workflow for modelling healthcare expenditure, rather than to derive nationally representative estimates for the US population. As such, all results should be interpreted as methodological illustrations.

The analytical methods applied to this dataset are described in the subsequent subsections. They are recommended for routine use in empirical practice and serve as a reminder that robust health policy analysis requires not only advanced modelling techniques, but also a structured, reproducible, and transparent analytic workflow that facilitates translation from evidence generation to implementation.

All data management and analyses were conducted using Stata MP V18 (Stata Corp, Texas, USA). Statistical significance was assessed at the 5% level (two-sided tests), unless otherwise specified.

### 3.1 Linear regression and its modifications

Linear regression models the relationship between a continuous outcome (Y) and predictors (X), estimating coefficients using Ordinary Least Squares (OLS) by minimizing squared residuals. Coefficients indicate direction and magnitude of association, with significance assessed via t-tests and overall fit evaluated by the *F*-test. Under Gauss–Markov conditions, OLS estimators are unbiased and efficient (11). When assumptions such as homoscedasticity are violated, robust regression using Iteratively Reweighted Least Squares (IRLS) can reduce the influence of outliers (12). For nonlinear structures, Nonlinear Least Squares (NLS) is applied (13). If functional form is unknown, kernel regression (e.g., Epanechnikov) offers flexible estimation.

### 3.2 Generalized linear model & finite mixtures model

Because total medical expenditure is non-negative, right-skewed, and often heteroscedastic, distributions from the exponential family (e.g., gamma) may provide better fit than the OLS. The Generalized Linear Model (GLM) extends linear regression by linking the mean of Y to predictors through a suitable link function (14). Parameters are estimated using Maximum Likelihood Estimation (MLE), which yields consistent and asymptotically efficient estimates. If latent subpopulations are present, a Finite Mixture Model (FMM) may improve fit, with estimation performed via the Expectation–Maximization (EM) algorithm (15).

### 3.3 Single & ensemble decision trees with bagging and boosting

Classification and Regression Trees (CART) is a non-parametric machine learning algorithm that predicts Y by recursively partitioning data into homogeneous subsets through binary splits (16). At each node, the optimal split minimizes variance in Y, measured by the regression sum of squares (RSS). Splitting stops when further reduction in RSS is negligible or when node size falls below a threshold. CART handles nonlinearity and interactions, performs automatic variable selection, accommodates outliers, and provides intuitive decision rules. Random Forest (RF) improves predictive accuracy by averaging multiple trees built from bootstrapped samples (17), reducing overfitting. Gradient Boosted Regression (GBR) builds trees sequentially, correcting prior errors iteratively for enhanced predictive performance (18). Both RF and GBR are ensemble learning techniques.

## 4 Analysis & results

The empirical findings were derived from the abovementioned quantitative methods. The primary objective is to identify the major predictors of medical expenditure in a consolidated and comparative manner. By synthesising results across models, the analysis strengthens the robustness of inference and reduces reliance on any single methodological approach. These findings provide actionable insights to inform evidence-based recommendations and support the future design and implementation of cost-containment strategies within Singapore's healthcare system.

### 4.1 Sample characteristics

The effective sample comprised 2,955 healthcare users (aged 65 years and above) with reported medical expenditure, with a mean age of 74.2 years. Approximately 58% were female and 59% were retirees. Total medical expenditure was highly right-skewed (mean: US$7,290.2; SD: 11,990.8; maximum: US$125,610), indicating substantial cost variability among older adults. More than 80% of participants had at least one chronic condition, and over 60% had multiple conditions. Over 59% had supplemented private insurance. About 29% had suffered injuries. In addition, 43.6% reported physical limitations and 28.8% reported activity limitations. These findings underscore the strong association between chronic disease burden, functional impairment, and elevated medical expenditure among the elderly population.

### 4.2 Empirical results

The OLS regression showed that the number of chronic conditions (*totchr*) was significantly associated with total medical expenditure (estimated *β* ≈ 1,814.40, *p* < 0.001). Other things being equal, a one-unit increase in the number of chronic conditions was associated with an approximate $1,844 increase in medical expenditure. Age, gender, injury, activity limitations, and physical limitations were also statistically significant. Diagnostic tests revealed heteroscedasticity and pronounced right-skewness in expenditure, prompting the use of heteroscedasticity-robust standard errors and robust regression to assess sensitivity. These analyses consistently confirmed the central role of chronic conditions. The robust regression estimated with IRLS also yielded a significant effect in *totchr* (estimated *β* ≈ 811.30, *p* < 0.001) after down-weighting the outliers. Compared with OLS, robust estimates were substantially smaller, indicating that a small number of high-cost observations had strongly influenced mean-based estimates.

To accommodate the strictly positive and right-skewed distribution of medical expenditures, a Gamma-distributed GLM with a log link was estimated. It confirmed the key finding that the number of chronic conditions remained a significant variable of medical costs.

FMM was subsequently applied to identify latent subpopulations. Based on Bayesian Information Criterion (BIC) minimisation, a parsimonious two-component model was selected, thus distinguishing a low-expenditure group from the high-expenditure group. The high-cost group comprised older adults, and individuals with more chronic conditions. This classification provides useful insights for designing targeted and cost-effective intervention strategies in Singapore.

Nonparametric kernel regression using the Epanechnikov kernel demonstrated that marginal medical costs increase progressively with the number of chronic conditions. This pattern was corroborated by the Exponential Mean Model (EMM), which suggests that medical expenditure may escalate nonlinearly, potentially rising exponentially once individuals reach a critical threshold of approximately three to four chronic conditions. These findings highlight the compounding financial impact of multimorbidity and underscore the importance of early preventive intervention to mitigate cost acceleration.

Building upon the statistical analyses, CART identified physical limitations and income as the primary splitting variables. However, compared with other methods, CART was more prone to overfitting and exhibited limited predictive stability. Reducing variance and demonstrating strong predictive performance, RF regression with 50 trees and 5-fold cross-validation identified income, activity limitation, age, and chronic conditions as key predictors of medical expenditure.

GBR achieved the highest overall predictive accuracy. It was implemented with Gaussian loss for the log-transformed expenditure outcome. The model was trained using 80% of the data, with 20% reserved for validation. A maximum of 1,000 boosting iterations were allowed. Stochastic gradient boosting was applied with a subsampling rate of 0.5 to reduce overfitting. Weak learners were restricted to depth-1 trees (additive specification without higher-order interactions). Variable importance measures were obtained using relative influence statistics. Three models were trained simultaneously, and the results were comparable to RF in identifying key cost drivers. A fixed random seed ensured reproducibility.

Robustness checks included implementing five-fold cross-validation, and applying elastic net regularization to address potential multicollinearity and overfitting. The elastic net approach, which combines L1 (lasso) and L2 (ridge) penalties, enabled simultaneous variable selection and coefficient shrinkage, thereby improving model generalizability (19). The regularisation strength and mixing parameter were identified with a grid search. Across all specifications, chronic conditions consistently emerged as the most important variable of medical expenditure, confirming the stability, robustness, and reliability of the findings.

### 4.3 Key findings and implications

A key finding appears emphasized by the analyses above: medical expenses come actively and substantially associated to the prevalence of chronic diseases. Furthermore, it examines how factors like activity limitations, physical limitations and income impact people's healthcare spending. Based on these empirical findings, one is able to apply relevant principles and knowledge drawn from behavioural medicine to enhance general and patient health outcomes while optimizing healthcare institution resources. It is important to emphasise that the above estimates reflect conditional associations rather than causal effects. Given the observational nature of the data, no claims of causality are made.

These findings may be relevant to future consideration of digital platform development and preventive-care planning in Singapore. Any such application would require local adaptation, stakeholder input, and separate evaluation of feasibility, acceptability, and effectiveness. A locally adapted pilot in an elderly community could be considered in future work before any broader scale-up is contemplated. Accordingly, the subsequent sections will examine how these findings could assist an ageing nation like Singapore in establishing a robust digital platform to better manage medical costs given the current societal conditions.

## 5 Major challenges, concerns & potential solutions

### 5.1 Singapore−an expensive liveable city

Singapore is widely recognised for its high standard of living, world-class education system, advanced infrastructure, and strong public safety. While this exceptional liveability attracts global businesses and talents, it also entails substantial social and financial costs. Consequently, the city-state is consistently ranked among the world's most expensive cities.

Several structural factors contribute to this high cost of living. Limited land availability, combined with strict zoning regulations, drives up real estate prices. Vehicle ownership is tightly regulated through the Certificate of Entitlement (COE) system, significantly increasing transportation costs. As a resource-scarce nation reliant on imports for food, fuel, and essential goods, Singapore remains vulnerable to global supply chain disruptions and price volatility. Economically, it functions largely as a price taker (20).

Decades of sustained economic development have resulted in a high-income economy with correspondingly high wages. Elevated disposable income can generate inflationary pressure on goods and services, while higher business operating costs are often passed on to consumers. Strict food safety standards and dependence on overseas supply further elevate food prices. Moreover, substantial investments in green initiatives, public transportation, and urban planning—while enhancing long-term sustainability and quality of life—contribute to higher upfront costs that influence the overall cost structure of the economy.

### 5.2 Challenges facing Singapore’s healthcare system

Public concern over rising healthcare costs has intensified in recent years. Singapore's healthcare system has long sought to balance affordability with high quality, achieving considerable success. However, sustaining a world-class system—supported by highly trained professionals, advanced facilities, and cutting-edge medical technologies—requires substantial investment (7). Strict regulatory standards, while essential for maintaining quality and safety, also contribute to compliance and operational costs.

Singapore relies heavily on imported medical equipment and pharmaceuticals, making healthcare expenditure sensitive to international pricing and export-related costs. Many medications are expensive due to high research, development, and regulatory expenses. Although Singapore's financing framework subsidises essential services within the public healthcare system, not all treatments are fully covered.

The expansion of private insurance has also influenced cost dynamics. While it increases access, it may encourage greater utilisation of medical services, contributing to expenditure growth (21). Additionally, as the population ages, demand for more complex and long-term care is expected to rise, further increasing healthcare spending.

Overall, Singapore faces mounting pressure from demographic ageing, imported medical inflation, and the ongoing need to balance affordability with quality. Despite continued policy efforts to maintain sustainability, healthcare costs are projected to increase, necessitating careful fiscal planning and strategic resource allocation (7).

### 5.3 Initiatives to ensure affordability and accessibility

The government has introduced a range of measures to strengthen preventive care, promote healthy living, and encourage early disease detection through health screenings, lifestyle programmes, and exercise incentives (7). Efforts have also been made to enhance insurance coverage and healthcare financing. MediShield Life—Singapore's national basic health insurance scheme—has been strengthened to cover essential treatments while maintaining affordable premiums. Singaporeans are encouraged to consider Integrated Shield Plans— optional private insurance plans built on top of MediShield Life—for private care. The usage of MediSave—Singapore's national medical savings scheme—has been expanded to cover preventive services and outpatient treatments, while discouraging excessive reliance on subsidies.

Targeted subsidies remain available for vulnerable groups. The Medication Assistance Fund supports access to essential drugs for low-income patients. The Community Health Assist Scheme (CHAS) subsidises general practitioner and dental visits. Vaccinations under national immunisation schedules are subsidised or fully covered for eligible individuals, including protection against hepatitis B, measles, pneumococcal disease, influenza, and varicella. COVID-19 vaccinations have also been provided free of charge to eligible residents. In addition, the government monitors and regulates medical fees to discourage excessive pricing in the private sector.

Overall, policy emphasis lies in ensuring affordability and accessibility while fostering cost-conscious public behaviour. The key challenge is how to cultivate appropriate health behaviours: promoting healthy ageing, improving medication adherence, and encouraging the use of cost-effective care options (22). Preventive care and community-level engagement are essential, requiring deeper understanding of patient behaviour and public perceptions towards healthcare systems and institutions.

These initiatives and recommendations are grounded in the empirical identification of key predictors of medical expenditure—most notably the prevalence of chronic conditions, income level, and physical limitations. The findings highlight that healthcare costs are not randomly distributed across the population but are concentrated among individuals with higher disease burden, lower socio-economic status, and functional impairments. As such, the emphasis on preventive care, targeted subsidies, and insurance design reflects an evidence-informed attempt to address structurally identifiable cost drivers on top of the broad policy options.

### 5.4 Promoting behavioural medicine with data science: opportunities

Effective cost containment must address two interrelated dimensions: (a) improving population and patient health, and (b) optimising institutional resource use. Rather than introducing entirely new frameworks, solutions should align with existing national programmes and relevant implementation science principles.

A central pillar is the Smart Nation initiative**,** launched in 2014 to harness digital technologies, data integration, and innovation to enhance quality of life and institutional efficiency (3). Within healthcare, Smart Nation has supported the integration of platforms such as HealthHub and the expansion of telehealth services, enabled by parallel advances in digital infrastructure, fintech, mobility systems, and the Internet of Things (IoT). This technological foundation creates opportunities to better anchor patients within community-based care, reduce avoidable hospital admissions, and strengthen preventive health interventions.

Community-centric care has been reinforced through step-down facilities, early discharge programmes, and home-based recovery models inspired by international experiences. Locally, the Healthy 365 programme, led by the Health Promotion Board (HPB), incentivises healthy lifestyles via app-based tracking, health points, and national campaigns. Complementing this is Healthier SG, launched in 2023 to shift care from reactive treatment to preventive, family physician-led management. Early outcomes indicate increased screenings and vaccination uptake within regional clusters.

As digital infrastructure matures, large-scale structured and unstructured health data will become increasingly available. The challenge lies not in technological capability but in governance: determining what data to capture, how to integrate and secure it, and how to translate analytics into timely interventions (23). Streaming analytics and complex event processing can support real-time monitoring and targeted escalation, improving community-based management and resource allocation.

Behavioural medicine is equally critical. Chronic diseases—such as diabetes, hypertension, and cardiovascular conditions—drive sustained costs (24). Interventions promoting healthy eating, physical activity, smoking cessation, medication adherence, stress management, and self-monitoring can prevent disease progression and reduce hospital utilisation. Community and workplace wellness programmes further strengthen early detection and public awareness. By integrating behavioural strategies with digital platforms and implementation science, Singapore can enhance preventive care while advancing sustainable medical cost containment.

### 5.5 Embracement of technology to facilitate behavioural medicine

The central question is how to effectively promote behavioural medicine initiatives within an increasingly digitalised Singapore. As healthcare delivery becomes more technology-enabled, it is critical to address behavioural, psychological, and structural barriers that may hinder adoption. Digital platforms alone do not guarantee behaviour change; user acceptance, trust, and usability remain decisive factors.

**Figure 1:**
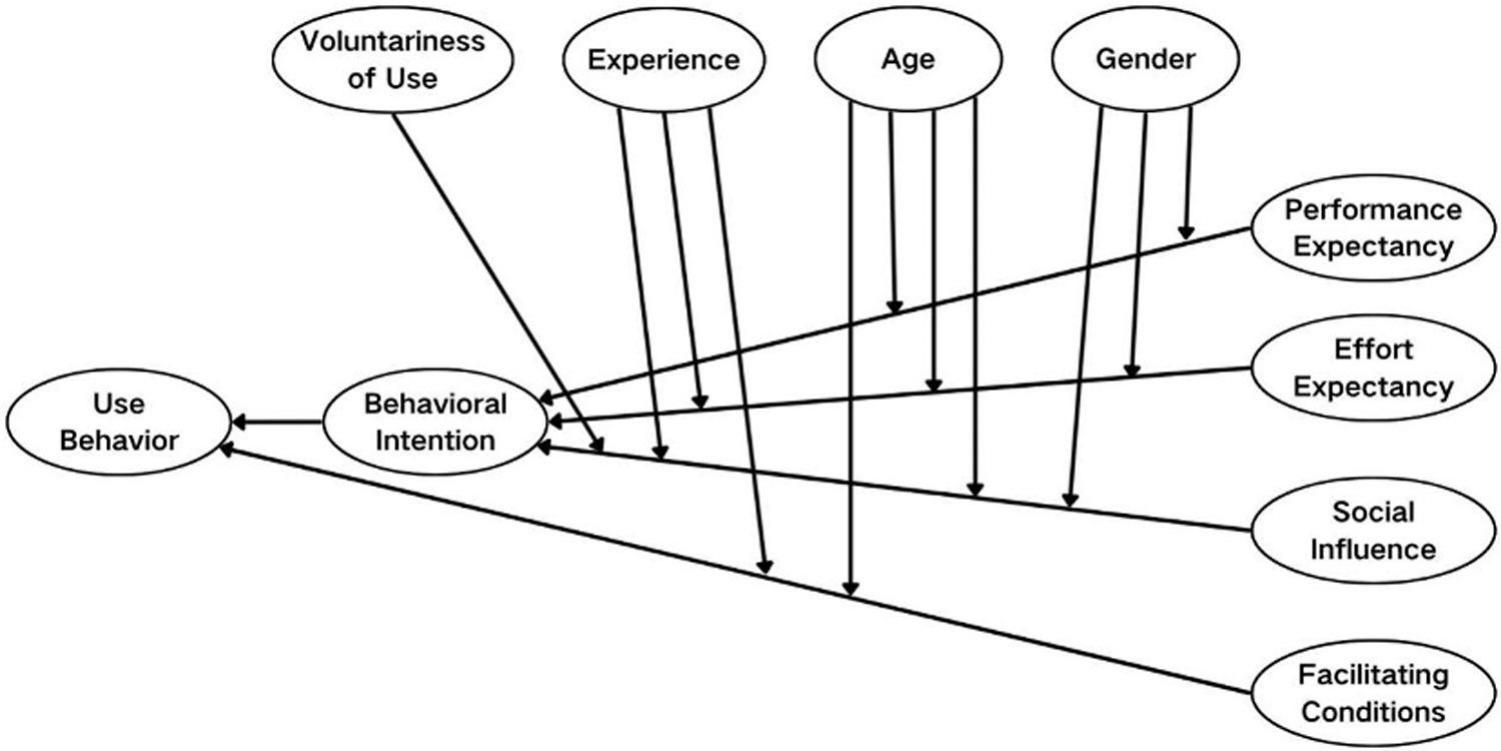
Conceptual framework of unified theory of acceptance and use of technology.

From a technology adoption perspective, the Unified Theory of Acceptance and Use of Technology (UTAUT) provides a conceptual framework for considering factors that may influence the uptake of digital health tools. The model identifies four core determinants—performance expectancy, effort expectancy, social influence, and facilitating conditions—that are theorised to shape behavioural intention and use behaviour, with demographic and experiential factors such as gender, age, experience, and voluntariness of use acting as moderators (25) (Figure 1). In this study, UTAUT is referenced as a theoretical lens for future implementation considerations rather than as a framework empirically tested in the analysis.

When applied to behavioural medicine, the framework helps explain how digital tools—such as health apps, telemonitoring systems, and AI-driven coaching—can support sustained behaviour change and improved health outcomes (26). By strengthening perceived usefulness, simplifying user experience, leveraging social norms, and ensuring adequate institutional support, policymakers can enhance digital uptake and maximise the impact of behavioural medicine initiatives.

Under Performance Expectancy, digital technologies such as mobile health applications and wearable devices enable patients to monitor key health indicators, including physical activity, sleep patterns, and medication adherence. Personalised insights and real-time feedback can strengthen patients’ confidence in self-management, thereby improving health outcomes. With respect to Effort Expectancy, user-friendly interfaces and intuitive designs reduce the cognitive and practical burden of using health technologies. Telehealth platforms that facilitate seamless communication between patients and providers simplify access to care and encourage sustained engagement. In terms of Social Influence, digital health platforms that incorporate online communities and peer networks can motivate behaviour change. Sharing experiences, providing mutual encouragement, and fostering accountability enhance commitment to healthier lifestyles. Finally, effective adoption depends on Facilitating Conditions, including accessible instructions, responsive technical support, reliable internet connectivity, and device compatibility. These infrastructural supports enable consistent integration of digital tools into daily health routines.

Collectively, technological readiness reduces barriers to timely interventions and strengthens the implementation of behavioural medicine. The success of large-scale community healthcare initiatives ultimately depends on coordinated adoption by policymakers, healthcare institutions, and the public.

### 5.6 Embracement of technology by family physicians

Family physicians are central to community-based care and sustainable cost containment. However, several barriers must be addressed for their role to be fully realised within the proposed framework.

A key challenge lies in public misconceptions about family medicine. It is often conflated with general practice, leading to the perception that family physicians manage only minor ailments and routine check-ups. This overlooks their expertise in chronic disease management—the primary driver of long-term medical costs. Many patients assume that specialist or hospital-based care is inherently superior, underestimating the value of comprehensive, continuous, and coordinated primary care. In practice, family physicians are well positioned to discourage delayed help-seeking behaviour and to promote the uptake of preventive services, including screenings, vaccinations, and lifestyle counselling (27).

The Singapore government has strengthened institutional support for family medicine through formal specialty accreditation, the rollout of Healthier SG, improved primary–tertiary integration, and expansion of community infrastructure. Recognition by the Ministry of Health aligns Singapore with regional peers and elevates professional standards (28).

Technology adoption further enhances the role of family physicians. With AI-driven risk stratification, data analytics, and telemedicine platforms, they can identify high-risk patients early, personalise care plans, and coordinate multidisciplinary management. As frontline providers, family physicians are well positioned to promote digital health acceptance, guide patients in telehealth use, and contribute data-informed insights for policy and research collaboration. Strengthening their technological capabilities reinforces their leadership in preventive, evidence-based, and cost-conscious community care.

### 5.7 Transferability boundaries and system adoption considerations

This study employs the US MEPS as an empirical platform to demonstrate a structured analytic workflow integrating statistical and machine learning approaches. The findings should therefore be interpreted as an illustration of a transferable methodological pipeline rather than as direct evidence of healthcare cost determinants in Singapore.

Transferability requires adaptation to Singapore's data environment. Unlike the US, Singapore does not maintain a nationally harmonised public panel equivalent to MEPS. Application would require integration of administrative claims, electronic health records, and survey data within existing governance constraints. Differences in coding standards, sampling frameworks, and longitudinal structure would necessitate recalibration of preprocessing and feature engineering procedures.

Institutional context further limits direct inference. The US system is characterised by heterogeneous private insurance and market-based pricing, whereas Singapore operates a mixed financing model comprising MediSave, MediShield Life, Integrated Shield Plans, and government subsidies. Price controls, benefit design, co-payment rules, and subsidy tiers differ substantially, implying that expenditure magnitudes and elasticity estimates are system-specific and not cross-nationally transferable.

Moreover, the present analysis demonstrates associations rather than causal effects and does not model Singapore-specific policy mechanisms, including subsidy calibration or Healthier SG reforms. Accordingly, parameter estimates from MEPS should not be interpreted as policy prescriptions.

Potential simultaneity and omitted variable bias cannot be fully ruled out as this study is based on observational survey data. Higher healthcare utilisation may increase the likelihood of diagnosing chronic conditions, thus introducing possible reverse causality. In addition, unobserved factors such as disease severity, health literacy, or health-seeking preferences may confound the above-mentioned associations. Accordingly, the findings should be interpreted as conditional associations rather than causal effects.

What is transferable is the analytic architecture: structured data harmonisation, comparative modelling across statistical and machine learning methods, robustness testing, and integration of quantitative outputs into an implementation-oriented decision framework. The contribution of this study lies in providing a reproducible workflow that can generate context-specific evidence when applied to locally derived datasets.

## 6 Discussion−smart medicine

This paper advocates that integrating data science with implementation science offers a strategic pathway for Singapore to contain rising healthcare costs, particularly those driven by chronic diseases. Statistical and machine learning analyses of the MEPS consistently identify chronic disease burden as the strongest predictor of total medical expenditure. This finding underscores the need to move beyond treatment-focused models towards prevention, behaviour change, and system-level technological transformation—hallmarks of “smart healthcare.” One possible future application would be to explore this workflow in a locally adapted pilot setting, such as an elderly community, while recognising that any wider implementation would require separate feasibility, adoption, and effectiveness evaluation.

Singapore's Smart Nation strategy provides an enabling environment for this transition. IoT technologies, wearable devices, telemedicine platforms, and AI-enabled systems allow real-time monitoring of chronic conditions and early intervention. Such integration has the potential to reduce avoidable hospitalisations, improve continuity of care, and enhance community-based management in future implementation contexts. Programmes such as Healthy 365 illustrate how behavioural incentives can be digitally embedded, although stronger integration with clinical systems remains an important opportunity for future implementation.

Real-time data streaming and predictive analytics enable timely alerts for family physicians, resource planning for hospitals, and rapid public health responses. AI-driven chatbots further extend care by offering triage, medication reminders, and behavioural coaching at scale, potentially reducing workforce strain.

Nevertheless, technological adoption—particularly among the elderly—remains a barrier. Community-level digital literacy initiatives and user-friendly design are essential. Equally critical are robust data governance, privacy safeguards, and public trust. Evidence generation through pilot trials, cost-effectiveness evaluation, and behavioural adoption models (e.g., UTAUT) will determine scalability.

Ultimately, the convergence of behavioural medicine, digital technology, and implementation science positions Singapore to build a preventive, patient-centric, and data-driven healthcare system capable of sustaining long-term cost containment.

Singapore does not have a single, nationally harmonised panel dataset equivalent to the US MEPS. Developing a nationwide system to consolidate health-expenditure and utilisation data could greatly strengthen evidence-based planning—but it must be designed to respect Singapore's strict data-protection and access controls. Replication would require integration of administrative datasets (e.g., public hospital discharge data, MediShield Life and Integrated Shield Plan claims, MediSave withdrawals) alongside national health surveys and socioeconomic indicators. Variable definitions would need reconstruction to reflect Singapore's financing architecture, including tiered subsidies, co-payments, deductibles, withdrawal limits, and CPF-linked savings mechanisms.

Importantly, Singapore's mixed financing model—universal catastrophic insurance combined with mandatory medical savings and regulated public-sector pricing—differs structurally from the predominantly market-based US system. As such, price elasticity, utilisation behaviour, and insurance effects observed in MEPS cannot be assumed to operate similarly under Singapore's cost-sharing and subsidy framework.

Accordingly, this study does not presume cross-national parameter equivalence. Instead, it proposes a transferable, system-adaptable analytic pipeline that can be implemented using Singapore-specific data to produce contextually grounded evidence for healthcare cost management and policy formulation. By strengthening data-driven decision-making, this approach ultimately aims to enhance public health outcomes for Singaporeans through more targeted prevention strategies, efficient resource allocation, and sustainable healthcare delivery.

## Data Availability

The original contributions presented in the study are included in the article/Supplementary Material, further inquiries can be directed to the corresponding author.
